# The Association Between Nurses’ Perceptions of Teamwork and Nurse and Patient Outcomes in Hospital Settings: A Systematic Review

**DOI:** 10.1155/jonm/3356709

**Published:** 2026-08-04

**Authors:** Farzaneh Miri, Alireza Hajizadeh, Masoud Arefnezhad, Razie Alipour, Jalal Saeidpour, Young Sook Roh, Edris Kakemam

**Affiliations:** ^1^ Healthcare Innovations, Faculty of Health Sciences and Medicine, Bond University, Gold Coast, Queensland, Australia, health.qld.gov.au; ^2^ National Institute for Health Research, Tehran University of Medical Sciences, Tehran, Iran, tums.ac.ir; ^3^ Department of Health Services Management, School of Public Health, Zabol University of Medical Sciences, Zabol, Iran, zbmu.ac.ir; ^4^ Non-Communicable Diseases Research Center, Research Institute for Prevention of Non-Communicable Diseases, Qazvin University of Medical Sciences, Qazvin, Iran, qums.ac.ir; ^5^ Red Cross College of Nursing, Chung-Ang University, 84 Heukseok-ro Dongjak-gu, Seoul 06974, South Korea, cau.ac.kr

**Keywords:** hospital, nurse outcomes, nurses, nursing, patient outcomes, teamwork

## Abstract

**Background:**

Teamwork has been recognized as a potentially important organizational factor associated with nurse‐ and patient‐related outcomes. Previous studies have been conducted to examine the association between nurses’ perceptions of teamwork and various outcomes in hospital settings. However, no systematic review has yet summarized this evidence. Therefore, this study aimed to summarize the available evidence on the association between nurses’ perceptions of teamwork and nurse and patient outcomes in hospital settings.

**Methods:**

This systematic review followed the Preferred Reporting Items for Systematic Reviews and Meta‐Analyses (PRISMA) guidelines. A comprehensive search was conducted using the keywords “teamwork,” “group work,” and “nurse,” along with their synonyms, across PubMed, Scopus, Web of Science, CINAHL, and the Google Scholar search engine. The quality of selected studies was assessed using the Critical Appraisal Skills Programme (CASP) checklists. The narrative synthesis method was used to summarize and classify the results.

**Results:**

A total of 33 studies met the inclusion criteria for this systematic review. The analysis identified two overarching themes: nurse‐related outcomes and patient outcomes. Twenty‐two studies (66.7%) reported that nurses’ positive attitudes toward teamwork were associated with more favorable levels in 17 nurse outcomes, which were categorized into four groups: (1) job attitudes and retention; (2) patient safety attitudes and competency; (3) occupational stress, fatigue, and conflict; and (4) ethical values and moral practice. Additionally, fourteen studies (42.4%) reported that such attitudes were linked to more favorable patient‐related outcomes, categorized as (1) nursing care processes; (2) adverse events reporting and occurrence; (3) quality and safety of care; and (4) patient‐centered and caring behaviors.

**Conclusion:**

Teamwork may be considered an important factor associated with more favorable nurse and patient outcomes. To promote collaboration, hospital administrators and nursing educators may consider implementing strategies to support teamwork within clinical settings. Nevertheless, further research employing longitudinal and interventional designs is necessary to clarify causal pathways and better understand the relationship between teamwork and these outcomes.

## 1. Introduction

The healthcare system is inherently complex and continuously evolving, making collaboration among nurses essential for ensuring patient safety and delivering high‐quality care [1, 2]. Nurses play a critical role not only in direct patient care but also in organizational and policy‐level processes that influence healthcare quality and outcomes [3, 4]. The World Health Organization (WHO) has recognized teamwork and team‐based care as key determinants of patient safety and quality of care [5]. Previous studies showed that appropriate attitudes toward teamwork among nurses may prevent turnover and shortages of nurses and other healthcare staff in hospitals [6, 7]. During and following the COVID‐19 pandemic, nurse turnover became a major challenge in healthcare organizations, particularly hospitals. Therefore, it is essential for nurse managers, hospital managers, and health policymakers to prioritize strengthening teamwork among nurses in clinical settings [8]. To better understand the importance of teamwork in healthcare, the integrative framework of teamwork, clinician occupational well‐being, and patient safety proposes mechanisms through which teamwork may influence clinician well‐being and patient safety, providing a comprehensive model for future empirical research.

Nurse outcomes reflect nurses’ work‐related experiences and occupational well‐being and include indicators such as job satisfaction, burnout, and turnover intention [9]. Existing systematic reviews have identified several factors influencing nurse outcomes, such as nurse staffing levels [9, 10] and working hours [11]. Prolonged working hours are associated with adverse nurse outcomes, including increased fatigue and reduced job satisfaction [11]. In hospital settings, higher patient‐to‐nurse ratios have consistently been associated with adverse nurse outcomes, including increased burnout, lower job satisfaction, and greater turnover intention [9, 10]. Moreover, studies evaluating the implementation of mandated minimum nurse‐to‐patient ratios have found associations with more favorable nurse outcomes [12]. However, findings regarding the association between these staffing policies and patient outcomes remain unclear.

Nurse‐sensitive patient outcomes are defined as patient outcomes that are directly influenced by nursing care and may include hospital‐acquired infections, falls, pressure ulcers, medication errors, and patient satisfaction [13, 14]. Systematic reviews have identified several influencing factors, including nurse staffing [13, 14], working conditions [15], leadership styles [16], and the overall work environment [13]. While relational leadership shows consistent positive associations with patient outcomes [16], the relationship between nurse working conditions and patient outcomes remains inconclusive [15]. Evidence suggests that adequate staffing is associated with a lower risk of patient falls and mortality [14], although the relationships between mandated nurse‐to‐patient ratios and patient outcomes remain mixed [12]. In addition, recent studies have emphasized the role of supportive work environments and an appropriate skill mix in improving the quality of care [13]. Multivariate analyses of Comprehensive Nursing Care Service Policy units show that nearly half of the associations with patient outcomes were positive and significant, despite methodological limitations [17]. Teamwork encompasses attitudes, behaviors, and cognitions. In this review, nurses′ attitudes toward teamwork were conceptualized broadly as nurses’ positive or negative evaluations, perceptions, and beliefs regarding teamwork within nursing units. Therefore, related constructs such as teamwork climate, perceived teamwork, nurse–nurse collaboration, team cohesion, and participative teamwork were considered conceptually aligned dimensions reflecting nurses’ perceptions and attitudes toward teamwork. Teamwork attitudes, defined as what team members feel or believe, can include concepts such as motivation and trust [7]. Healthcare researchers in various disciplines are increasingly interested in teamwork processes in healthcare settings. In this regard, previous studies have investigated the associations between teamwork and performance outcomes [8, 9]. The level of teamwork in different conditions depends on several factors, and the workplace in hospitals is one of these factors [10]. For example, within hospitals, nursing personnel working in intensive care units reported more positive perceptions of teamwork than those working in medical‐surgical units [11]. Several tools have been introduced to measure the level of teamwork, one of the most famous of which is the Teamwork Attitudes Questionnaire (TAQ) [12]. This instrument was developed and introduced under the auspices of the Team Strategies and Tools to Enhance Performance and Patient Safety (TeamSTEPPS) initiative, a program sponsored by the Agency for Healthcare Research and Quality [13].

Nurses make up a significant portion of the global healthcare workforce, especially in hospital settings, and positive teamwork among nurses is linked to better multidisciplinary teamwork and favorable healthcare outcomes [18]. Previous evidence has shown that positive teamwork among nurses is an important factor associated with patient‐centered care (PCC) in hospitals [19]. Also, positive attitudes and perceptions toward teamwork are the initial crucial steps in guaranteeing the provision of safe and high‐quality patient care [20]. Evidence suggests that teamwork is associated with increased nursing productivity, greater job satisfaction, reduced stress among nurses, better patient safety outcomes, higher patient satisfaction, and higher quality of care [21]. In addition, supportive organizational environments have been linked to stronger organizational commitment among healthcare staff [22].

Although prior systematic reviews have examined the associations between nurse working conditions and nurse‐sensitive patient outcomes [11, 15, 17], as well as the association between nurse staffing levels and both nurse and patient outcomes [12, 14], notable limitations persist. These reviews have predominantly focused on structural determinants such as staffing levels and working hours, with insufficient attention given to relational or process‐oriented factors like teamwork. Furthermore, the evidence supporting the link between nurse working conditions and patient outcomes remains inconclusive [15], and the effects of mandated staffing policies continue to yield inconsistent findings [12]. Individual studies suggest that teamwork may be positively associated with nurse outcomes and the quality of patient care through better communication, coordination, and mutual support. However, a comprehensive synthesis of the empirical evidence linking teamwork to both nurse outcomes and nurse‐sensitive patient outcomes is still lacking. Given the fragmented and heterogeneous nature of current research, largely due to methodological and contextual variations, there is a critical need for an integrated systematic review.

The integrative framework of teamwork, clinician occupational well‐being, and patient safety in hospital settings provides a comprehensive model for understanding the mechanisms through which teamwork influences clinician well‐being and patient safety [23]. The integrative framework of teamwork, clinician occupational well‐being, and patient safety in hospital settings provides a comprehensive model for understanding the relationships among teamwork, clinician well‐being, and patient safety. Despite growing interest in teamwork within healthcare settings, evidence regarding its association with nurse outcomes and nurse‐sensitive patient outcomes remains fragmented and heterogeneous. Therefore, a comprehensive synthesis of the available evidence is needed to clarify these relationships and inform clinical practice, policy development, and future research. Accordingly, this systematic review aimed to synthesize the available evidence on the association between nurses’ perceptions toward teamwork and both nurse outcomes and nurse‐sensitive patient outcomes in hospital settings.

## 2. Materials and Methods

### 2.1. Study Design

This study is a systematic review conducted in accordance with the Preferred Reporting Items for Systematic Reviews and Meta‐Analyses (PRISMA) guidelines [24]. The guiding research question of this review was “What is the association between nurses” perceptions of teamwork and nurse and patient outcomes in hospital settings?

### 2.2. Source of Data and Search Strategy

To identify relevant studies, the databases Medline (PubMed), Scopus, the Cumulative Index to Nursing and Allied Health Literature (CINAHL), and Web of Science (WoS) were searched using predefined keywords. Boolean operators were used to combine keywords and retrieve all potentially eligible studies. Keywords were selected broadly to ensure no restrictions in study identification. The final search string was (“teamwork” OR “interprofessional relations” OR “group work” OR “cooperative behavior”) AND (“nurses” OR “nursing staff”). Medical Subject Headings (MeSH) terms and Title/Abstract fields were used to refine the search. Although the same core search concepts were applied across all databases, the search strategy was adapted to the indexing terms and search functionalities of each database. The complete database‐specific search strategies are presented in Supporting File 1. The search strategy was developed by the authors in consultation with an expert librarian. In addition, Google Scholar was used to identify recently published studies, and the reference lists of included studies were manually screened. An updated search was conducted on 15 April 2026.

### 2.3. Inclusion and Exclusion Criteria

Studies were included in this review if they met the following criteria:

1. Examined nurses’ perceptions of teamwork in hospital settings as the independent variable and investigated its association with nurse and/or patient outcomes. This also included studies assessing related constructs such as teamwork climate, collaboration, cohesion, participative teamwork, and perceived teamwork.
2. Were published in English‐language, peer‐reviewed academic journals.
3. Reported outcomes related to nurses and/or patients.
4. Used validated or standardized instruments to measure teamwork and related variables, such as the Nursing Teamwork Survey (NTS), the TeamSTEPPS Teamwork Attitudes Questionnaire (T‐TAQ), or other validated tools developed by reputable organizations and applied in hospital settings.

Also, studies were excluded if they met any of the following criteria:

1. Investigated multidisciplinary or interprofessional teamwork involving physicians, nurses, and other healthcare professionals, as the focus of this review was specifically on nurses’ teamwork attitudes in hospital settings.
2. Focused on teamwork models or approaches in educational settings rather than clinical hospital environments.
3. Were case reports, case series, reviews, or editorials.
4. Used qualitative designs due to the lack of quantifiable evidence on the association between nurses’ teamwork attitudes and outcomes.
5. Did not report clear or extractable data on the relationship between nurses’ perceptions of teamwork and relevant outcomes in hospital settings.

### 2.4. Study Selection Process

Two reviewers (E.K. and F.M.) independently screened the titles and abstracts of the identified studies. Subsequently, they assessed the full texts of potentially eligible studies and selected those that met all inclusion criteria. Any discrepancies between the two reviewers were resolved through discussion and consensus, with the involvement of a third reviewer (A.H.) when necessary.

### 2.5. Data Extraction

A standardized data extraction form was designed for data extraction by the authors. The details of data extraction from the selected studies comprised the first author, year of publication, country, design, sample size, hospital type and unit, assessment tool used for nurses’ teamwork, outcomes measured, and main findings related to the teamwork of nurses in hospitals. Two reviewers piloted the initial data extraction form on the first five studies to assess its appropriateness, accuracy, and relevance. Data from each study were extracted independently by two authors. Disagreements between authors regarding the selection of studies were resolved by discussion and referral to the third author.

### 2.6. Quality Assessment of Studies

Methodological quality of the included studies was assessed with the 12‐item Critical Appraisal Skills Program (CASP) checklists [25]. Each item was rated as “Yes,” “No,” or “Cannot tell.” The total appraisal scores were then converted into percentages. Studies scoring ≥ 70% were categorized as good quality according to CASP criteria: those scoring 30%–70% were considered of moderate quality, and those scoring ≤ 30% were considered low quality (weak).

### 2.7. Data Analysis

Meta‐analysis was not performed due to heterogeneity in the definition and measurement of outcomes, as well as differences in methodological approaches used to collect outcome data. Therefore, the extracted data were synthesized descriptively in accordance with the Synthesis Without Meta‐analysis (SWiM) guidelines [26]. The SWiM guidelines were applied to guide study grouping, criteria for synthesis, synthesis methods, data presentation, and reporting of synthesis findings. This approach was used to structure and report both consistent and conflicting findings in the present review. In this systematic review, findings regarding the association between nurses’ attitudes toward teamwork and nurse and patient outcomes were narratively synthesized and presented in a categorized format with the involvement of the research team. All outcomes were inductively identified and organized into categories based on their content.

## 3. Results

### 3.1. Search Results

**FIGURE 1:**
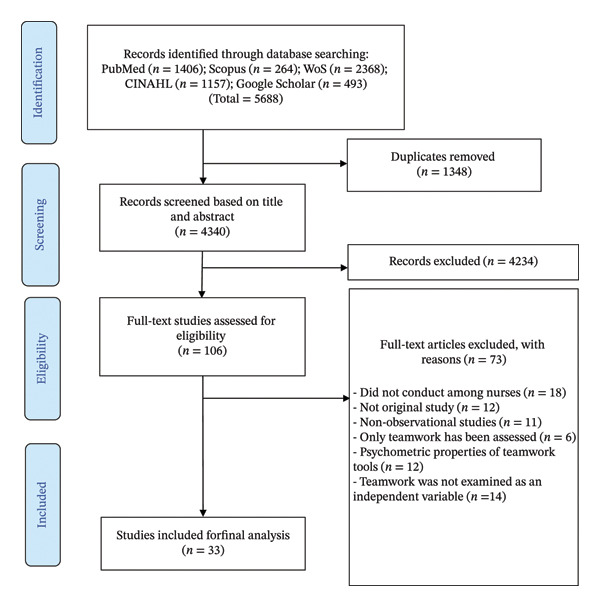
PRISMA flow diagram of the literature screening process.

The initial search identified 5688 records from various sources. After removing duplicates and screening titles and abstracts, 106 studies were assessed for eligibility through full‐text review. Finally, 33 studies met the inclusion criteria and were included in the evidence synthesis. The study selection process is presented in Figure 1.

### 3.2. Characteristics of the Included Studies

The included studies were published between 2008 and 2026, and most of the studies (16 studies; 48.5%) were carried out in the United States [21, 27–33] and Iran [34–41]. In addition, four studies were conducted in South Korea [19, 2–44] and three studies and two studies were conducted in Turkey [45–47] and Jordan [6, 48], respectively. Other studies were conducted in countries such as Saudi Arabia [49], the United Kingdom [50], China [51], Belgium [31], Eswatini [52], Taiwan [53], France [54], and Iceland [8]. Also, a collaborative study was carried out in Belgium, Italy, France, the United Kingdom, Slovakia, Norway, Finland, Germany, the Netherlands, and Poland [55]. In total, 154,840 nurses participated in the 33 included studies. Across all included studies, various tools were used to assess the attitude of hospital nurses toward teamwork. The most used tool for collecting data about nurses’ attitudes toward teamwork was the T‐TAQ and NTS scales, which were used in 15 studies (46.45%) and 10 studies (30.3%), respectively. Although the included studies used different instruments, all measures assessed nurses’ evaluations or perceptions of teamwork and were therefore grouped under the overarching concept of attitudes toward teamwork for the purpose of this review. The majority of studies were single‐center studies and were conducted in teaching hospital settings. 19 studies [6, 8, 21, 27, 28, 31–33, 36, 37, 40, 43, 44, 46, 47, 49, 53–55] nurse outcomes associated with teamwork and 11 studies [19, 29, 30, 34, 35, 41, 42, 45, 48, 50, 52] examined only patient outcomes, whereas three studies [38, 39, 51] assessed both nurse and patient outcomes related to teamwork. The characteristics of the included studies are presented in Supporting File 2.

### 3.3. Quality Assessment of Included Studies

Based on our CASP appraisal, 29 of the 33 included studies (87.9%) met at least 70% of the appraisal criteria and were categorized as good quality, while 4 studies (12.1%) met 30%–70% of the criteria and were categorized as moderate quality. No studies were categorized as poor quality, and none were excluded based on quality appraisal scores. However, these ratings should be interpreted cautiously. Although most included studies met many CASP appraisal criteria, the evidence base should not be interpreted as methodologically strong overall. The predominance of cross‐sectional, observational, and self‐reported designs means that important risks of bias remain, including residual confounding, reverse causality, selection bias, and common‐method bias.

### 3.4. Nurses’ Perceptions of Teamwork

Across the reviewed studies, nurses’ teamwork scores were generally reported at moderate to relatively high levels (Supporting File 2). On 5‐point rating scales, most scores ranged from approximately 3.0 to 4.3, reflecting acceptable to favorable perceptions of teamwork among nurses. These findings suggest that teamwork was generally perceived as satisfactory across the included study settings. A few studies reported lower scores (around 1.89–2.84) [27], suggesting variability across settings. Studies using different scale ranges showed comparable trends, with scores of 4.99 out of 7 [53] and approximately 2.6–2.83 out of 4 also reflecting moderate to high levels [27]. Findings are presented as observed associations rather than causal effects due to the cross‐sectional nature of the included studies.

### 3.5. Outcomes Related to Nurses’ Perceptions of Teamwork

**FIGURE 2:**
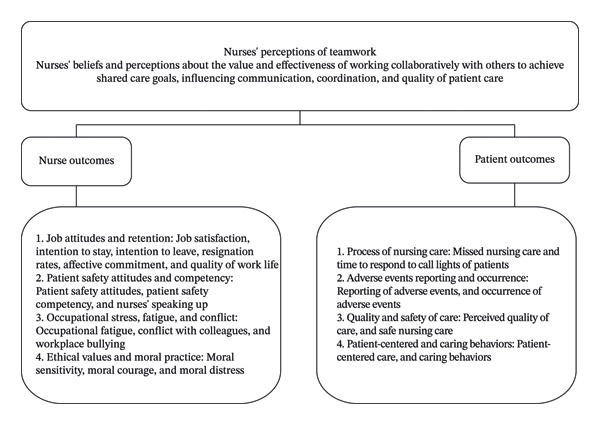
Outcomes associated with nurses’ perceptions of teamwork in hospital settings.

For data analysis, the findings of the included studies were organized into two main themes to facilitate a clearer understanding of the evidence: (1) nurse and patient outcomes assessed in the studies and (2) key findings related to nurses’ attitudes toward teamwork. The relationships between nurses’ attitudes toward teamwork and both nurse and patient outcomes are summarized in Table 1 and illustrated in Figure 2.

**TABLE 1:** Associations of nurses’ perceptions of teamwork and nurse and patient outcomes in included studies.

Category	Outcomes	Direction	Main findings
Nurse outcomes	Job satisfaction	Positive	*r* = 0.491; *p* < 0.01 [48], *p* < 0.001 [34], *r* = 0.297; *p* < 0.001 [31], OR = 4.63; *p* < 0.001 [8], OR = 1.896; *p* < 0.001 [28], (*β* = 0.944; *p* < 0.01) [44]
	Patient safety competency	Positive	(*r* = 0.231; *p* < 0.001 [38], *r* = 0.349 to 0.498; *p* < 0.001 [37], and *r* = 0.389; *p* < 0.001 [44]
	Intention to leave	Negative	*p* < 0.001 [34], *r* = −0.135; *p* = 0.043 [31], (Adj. OR = 3.65 to 10.88; *p* < 0.01) [55], (*β* = −0.941; *p* < 0.01) [44]
	Patient safety attitudes	Positive	*β* = 0.78, *p* < 0.01 [54], *r* = 0.451; *p* < 0.01 [50]
	Moral sensitivity	Positive	*r* = 0.48; *p* < 0.001 [40] and *r* = 0.653; *p* < 0.001 [52]
	Intention to stay	Positive	*r* = 0.587; *p* < 0.01 [6]
	Resignation rates in hospital	Negative	*β* = −2.92; *p* < 0.05 [27]
	Conflict with colleagues	Negative	*r* = 0.437; *p* < 0.01 [48]
	Safety climate	Positive	*r* = 0.841; *p* < 0.01 [6]
	Quality of work life	Positive	*r* = 0.334; *p* < 0.01 [55]
	Moral courage	Positive	*r* = 0.49; *p* = 0.002 [39]
	Occupational fatigue	Negative	*β* = −3.28 to −1.99; *p* < 0.001) [33]
	Moral distress	Negative	*r* = 0.531; *p* < 0.001 [41]
	Workplace bullying	Negative	*r* = −0.186 to −0.250; *p* < 0.05 [32]
	Nurses’ speaking up for unprofessional behaviors	Positive	*β* = 0.22; *p* < 0.05 [47]
	Nurses’ speaking up for patient safety concerns	Positive	*β* = 0.46; *p* < 0.01 [47]
	Affective commitment	Positive	*r* = 0.168; *p* = 0.017 [31]

Patient outcomes	Missed nursing care	Negative and not significant	*r* = −0.44; *p* < 0.001 [40], *r* = −0.29; *p* < 0.001 [35], *r* = −0.37; *p* < 0.01 [29], *r* = −0.084; *p* = 0.26 [52]
	Reporting of adverse events	Positive	OR = 2.12; *p* = 0.001 [43], Nurses who demonstrated appropriate reporting of AEs consistently exhibited a higher overall teamwork score than those whose reports were deemed inappropriate (*p* < 0.05) [35]
	Patient‐centered care	Positive	*β* = 0.32; *p* < 0.001 [19], *β* = 0.236; *p* < 0.05 [53]
	Perceived quality of care	Positive	*r* = 0.699; *p* < 0.001 [51]
	Caring behaviors	Positive	*r* = 0.295; *p* = 0.01 [46]; *r* = 0.555; *p* < 0.001 [42]
	Time to respond to call lights of patients	Not significant	No statistically significant relationship was observed between call light response time and teamwork [30]
	Occurrence of adverse events	Negative	Teamwork among nurses was significantly and negatively associated with the occurrence of AEs (*p* < 0.001) [35] and patient falls (*r* = −0.248; *p* < 0.001) [48]
	Safe nursing care	Positive	*r* = 0.59; *p* < 0.001 [39]

*Note: r*, Pearson versus Spearman correlation coefficient; *β*, standardized regression coefficient.

Abbreviation: OR, odds ratio.

#### 3.5.1. Nurse Outcomes

The majority of the included studies (22 studies, 66.7%) examined the relationships between nurses’ attitudes toward teamwork and 17 nurse‐related outcomes. These outcomes encompassed several domains, including job attitudes and retention factors (e.g., job satisfaction, intention to stay or leave, resignation rates, affective commitment, and QWL); patient safety attitudes and competencies (e.g., patient safety awareness and willingness to speak up about safety concerns); occupational stressors (e.g., fatigue, conflicts with colleagues, and workplace bullying); and ethical values and moral practice (e.g., moral sensitivity, moral courage, and experiences of moral distress).

##### 3.5.1.1. Job Attitudes and Retention Factor

Job satisfaction, a key indicator of nurses’ job attitudes, was examined in six studies. All six studies reported a statistically significant positive association between nursing teamwork and job satisfaction, suggesting that more favorable attitudes toward teamwork are linked to higher levels of job satisfaction among nurses [8, 21, 28, 31, 44, 47]. This finding reported a high level of consistency across studies, as all six investigations reported a positive association between teamwork and job satisfaction. Intention to leave (ITL) was investigated in four studies, which suggested that positive attitudes toward teamwork were associated with lower nurse turnover intentions [21, 31, 44, 55]. Additionally, one study conducted in Jordan reported a strong positive correlation between nurses’ attitudes toward teamwork and their intention to stay in their current positions, suggesting that favorable perceptions of teamwork may be related to higher levels of nurse retention [6]. Resignation rates were also shown to decrease with positive teamwork attitudes in a U.S. study [27]. Furthermore, teamwork was positively associated with affective commitment [31] and QWL [54] among hospital nurses.

##### 3.5.1.2. Patient Safety Attitudes and Competency Factor

Three studies, including those conducted in South Korea and Iran, consistently identified teamwork was significantly associated with patient safety competency. The findings indicated that more positive attitudes toward teamwork were associated with higher levels of patient safety competency among nurses, highlighting the important role of teamwork in promoting safe and high‐quality patient care [36, 37, 43]. The consistency of findings across all included studies strengthens the evidence supporting the relationship between teamwork and patient safety competency. Positive correlations between teamwork climate and patient safety attitudes were found in studies conducted in Taiwan and Saudi Arabia [49, 53]. A strong positive relationship between safety climate and teamwork was also observed among Jordanian nurses [6]. Moreover, one study highlighted the role of teamwork in encouraging nurses to speak up about unprofessional behaviors and patient safety concerns [46].

##### 3.5.1.3. Occupational Stress, Fatigue and Conflict Factor

Occupational fatigue and conflicts with colleagues were found to be inversely associated with positive attitudes toward teamwork. Specifically, a study conducted in Turkey reported a significant negative relationship between nurses’ attitudes toward teamwork and the frequency of conflicts with colleagues, indicating that more favorable perceptions of teamwork were associated with fewer interpersonal conflicts in the workplace [47]. Two studies found that teamwork was linked to fewer instances of workplace bullying [32] and was associated with lower occupational acute and chronic fatigue [33].

##### 3.5.1.4. Ethical Values and Moral Practice Factors

Two studies reported that teamwork has a positive relationship with moral sensitivity [39, 51], while one Iranian study reported a negative correlation between nurses’ attitudes toward teamwork and moral distress [40]. Nursing teamwork was also positively associated with moral courage in hospital settings [38].

#### 3.5.2. Patient Outcomes

Fourteen studies (42.4%) examined the association between teamwork and patient outcomes. These outcomes were grouped into four categories: (1) nursing care processes, including missed nursing care and response times to patients’ call lights; (2) adverse events (AEs) reporting and occurrence, encompassing both the reporting and frequency of AEs; (3) quality and safety of care, including perceived quality of care and the provision of safe nursing care; and (4) patient‐centered and caring behaviors, such as PCC and nurses’ caring behaviors.

##### 3.5.2.1. Nursing Care Processes Factor

Missed nursing care was examined in three studies, all of which reported lower levels of missed nursing care in hospitals where nurses demonstrated more positive attitudes toward teamwork. These findings suggest that favorable perceptions of teamwork may be related to care delivery processes and a lower occurrence of omitted nursing activities [29, 34, 39]. However, one study in China found no correlation between missed nursing care and teamwork [51]. Additionally, one study found no significant association between teamwork attitudes and time to respond to patients’ call lights [30]. Evidence regarding missed nursing care was generally supportive but not entirely consistent, as three studies reported significant inverse associations, whereas one study found no statistically significant relationship.

##### 3.5.2.2. Adverse Events Reporting and Occurrence Factor

Two studies from South Korea [42] and Iran [35] reported that positive teamwork attitudes were associated with higher reporting of AEs and lower occurrence of AEs in hospital settings [35]. Similarly, a study conducted among Jordanian nurses reported a significant negative correlation between the quality of nursing teamwork and the incidence of patient falls. This finding suggests that higher‐quality teamwork may be related to a lower occurrence of patient falls and better patient safety outcomes [48].

##### 3.5.2.3. Quality and Safety of Care Factor

A higher perceived quality of teamwork was positively associated with a higher perceived quality of care. This finding suggests that nurses who reported more favorable teamwork experiences were also more likely to perceive the quality of care delivered in their units as higher [50], and positive attitudes toward teamwork were linked to safe nursing care [38].

##### 3.5.2.4. Patient‐Centered and Caring Behaviors Factor

Nursing teamwork was significantly and positively associated with PCC in studies conducted in South Korea [19] and Eswatini [52]. Furthermore, positive teamwork attitudes were associated with more favorable caring behaviors [41, 45].

## 4. Discussion

Nurses hold a pivotal position within healthcare teams, and their perceptions of teamwork have been linked to various nurse and patient outcomes. The current study aimed to synthesize the literature examining the association between nurses’ perceptions of teamwork and nurse and patient outcomes in hospital settings. The findings of the current systematic review indicated that nurses’ positive teamwork perceptions were associated with more favorable nurse and patient outcomes in hospital settings. Despite these observed associations, the findings should be interpreted with caution, as the predominance of cross‐sectional studies in the reviewed literature does not allow causal relationships to be established. Overall, the evidence was most consistent for job satisfaction, ITL, patient safety competency, missed nursing care, and PCC, as these outcomes were supported by findings from multiple independent studies conducted across different countries and settings. In contrast, evidence for outcomes such as moral courage, QWL, affective commitment, and call light response time was limited because these outcomes were examined in only one or two studies. The findings should be interpreted cautiously because most included studies used cross‐sectional designs and self‐reported measures. Consequently, observed associations may be influenced by residual confounding, reverse causality, and common‐method bias. Positive teamwork perceptions may be related to better outcomes, but favorable outcomes may also influence nurses’ perceptions of teamwork. Likewise, evidence was consistent for several key outcomes, whereas evidence for some outcomes remained limited because they were examined in only a few studies.

Overall, despite some inconsistencies across studies and the absence of reported teamwork scores in several investigations, the findings indicate that nurses generally perceive teamwork as moderately strong. However, challenges remain in certain clinical and organizational contexts. The frequent use of the T‐TAQ and the NTS across studies suggests a reliance on well‐established, validated, and standardized instruments for assessing nurses’ perceptions and attitudes toward teamwork [56, 57]. The use of instruments with established reliability and validity is essential to ensure accurate and consistent measurement, thereby enhancing the credibility and comparability of findings. The T‐TAQ is commonly used because of its alignment with the TeamSTEPPS framework, whereas the NTS is designed to assess teamwork within nursing‐specific contexts. Future research should focus on the cross‐cultural validation of these instruments and evaluate their responsiveness to changes following teamwork interventions across diverse clinical settings.

The findings of this review highlight the important association between nurses’ positive perceptions of teamwork and favorable nurse‐related outcomes in hospital settings. Positive perceptions of teamwork were consistently associated with higher levels of job satisfaction, QWL, moral sensitivity, moral courage, organizational commitment, and intention to remain in the workplace. These outcomes are important indicators of workforce stability and a supportive and ethical work environment. In addition, more positive perceptions of teamwork were associated with better perceptions of the safety climate and more favorable patient safety attitudes [58]. Although the predominance of cross‐sectional studies limits causal inferences, the findings suggest that positive perceptions of teamwork may be an important organizational factor associated with nurses’ well‐being and patient safety culture and quality care. Most outcomes included in this review were measured using self‐reported questionnaires, including teamwork perceptions, job satisfaction, patient safety attitudes, caring behaviors, and missed nursing care. Therefore, common‐method bias may have inflated some reported associations. Relatively fewer studies examined more objective outcomes such as AE occurrence, patient falls, resignation rates, and call light response times.

This finding is consistent with previous research, which has shown that interprofessional teamwork is positively associated with job satisfaction [59]. Moreover, a qualitative study conducted within emergency departments revealed that positive perceptions of teamwork among healthcare professionals were associated with higher job satisfaction [60]. Higher‐quality communication among team members was associated with greater job satisfaction among nurses [31]. Furthermore, international study findings suggest that teamwork may mitigate staff turnover, reduce nurses’ intentions to leave, and decrease resignation rates within hospital settings [7, 55]. This finding is particularly important in the Iranian context, where turnover intention among hospital nurses has been reported as a substantial workforce challenge, highlighting the importance of organizational factors associated with nurse retention [61].

Furthermore, these findings are consistent with a previous review reporting that positive perceptions of teamwork were associated with staff well‐being outcomes, including lower levels of emotional exhaustion and burnout, as well as higher job satisfaction and organizational commitment. These factors may, in turn, be related to clinicians’ capacity to deliver safe patient care [62]. There is a need for updated and robust evidence to examine the relationships between nurse teamwork and a range of variables in clinical settings. The protection of nurses in the hospital is emphasized by promoting a teamwork culture. Brunault et al. [54] found that participative teamwork among nurses was associated with higher QWL, and this relationship was mediated by higher perceived organizational support and perceived organizational justice [54]. Furthermore, a study conducted among hospital physicians and nurses found that higher levels of teamwork were associated with better professional quality of life [63].

Conversely, negative perceptions of teamwork were associated with less favorable outcomes, including higher ITL, increased resignation rates, occupational fatigue, workplace bullying, and interpersonal conflict. The findings suggest that perceptions of teamwork may be related to nurse retention and are consistent with previous research reporting associations between supportive work environments, higher job satisfaction, and lower turnover intentions [64]. In the context of increasingly competitive nursing labor markets, fostering a positive teamwork culture may be an important consideration for healthcare organizations seeking to support nurse retention [27]. This review suggests that positive teamwork perceptions are associated with higher job satisfaction, stronger organizational commitment, and more favorable work‐related outcomes among nurses. These findings highlight the potential value of supporting collaborative team environments through communication, mutual support, and leadership initiatives. Positive teamwork was also associated with lower levels of occupational fatigue, moral distress, and workplace bullying, although further longitudinal and interventional research is needed to clarify these relationships. Prior qualitative studies support that teamwork helps manage fatigue [65, 66], while team‐building interventions enable nurses to recognize and address bullying through assertive communication [67]. These findings suggest that positive perceptions of teamwork are associated with better nurse well‐being and organizational outcomes. Healthcare organizations may benefit from supporting collaborative team environments through initiatives such as team training, communication strategies, and leadership development. However, given the predominance of cross‐sectional evidence, the direction and mechanisms of these relationships remain unclear, highlighting the need for longitudinal and interventional research. Interpretation of effect estimates should be undertaken cautiously because several studies reported unadjusted correlations, whereas others used multivariable regression models or adjusted odds ratios. Consequently, the strength of evidence varies across outcomes, and direct comparison of effect sizes is not appropriate.

Results showed that 14 studies (42.4%) examined the relationship between nurses’ perceptions of teamwork and patient‐related outcomes, including AEs reporting, missed nursing care, PCC, perceived care quality, caring behaviors, call light response time, and safe nursing care. Positive perceptions of teamwork were associated with lower levels of missed nursing care [29, 34, 39], increased AE reporting [35, 42], and decreased AE occurrence [35]. These findings suggest that positive teamwork attitudes may be associated with favorable patient safety and care quality outcomes. The findings suggest that positive teamwork is associated with more consistent completion of nursing care tasks, greater openness in reporting AEs and near misses, and more favorable perceptions of patient safety. These associations may reflect the role of collaborative and supportive work environments in facilitating communication and care delivery. Consistent with previous research, stronger teamwork was also associated with fewer missed care activities and more favorable safety‐related outcomes [68]. Therefore, a robust meta‐analysis focusing on teamwork’s effects on AE occurrence and reporting among nurses is warranted. Conversely, poor teamwork and communication failures are well‐documented factors associated with clinical errors [69], and a mixed‐methods systematic review highlighted the association between teamwork and missed care in acute settings [70].

The consistent associations between positive teamwork and more favorable PCC [19, 52], higher perceived quality of care [50], more positive caring behaviors [45], and safer nursing care [38] indicate that collaborative team environments support not only the technical aspects of nursing but also its relational and ethical dimensions. Positive perceptions of teamwork among nurses were associated with greater engagement, responsiveness, and empathy, as well as more favorable patient experiences and safety‐related outcomes. These findings highlight the potential value of teamwork in supporting holistic, high‐quality care. Furthermore, the review suggests that nurses’ positive attitudes toward teamwork are associated with more favorable perceptions of care quality and patient safety in hospital settings, including outcomes related to AEs [62]. Teamwork development may be considered as one component of broader quality and safety initiatives, although its causal contribution requires further evaluation [71], and complementary factors—such as interprofessional collaboration and hospital‐wide quality improvement initiatives—also play important roles in enhancing these outcomes.

Our findings suggest that positive nursing teamwork is associated with better PCC, perceived quality of care, caring behaviors, and safe nursing care. These findings are consistent with previous studies reporting associations between teamwork, patient safety, and care quality. Collectively, the evidence highlights the potential importance of teamwork in supporting positive patient care outcomes in clinical settings [50, 60]. An integrative review further emphasized that interdisciplinary teamwork and environments that foster open communication are essential prerequisites for PCC [72]. PCC emphasizes information exchange and shared decision‐making among patients, families, and healthcare providers [73], which likely explains the strong link between nursing teamwork and PCC. Positive perceptions of teamwork may facilitate the sharing of expertise and collaborative care planning, which can support well‐coordinated care and PCC. In addition, mutual respect and open communication among nurses are associated with more positive nurse–patient interactions. Overall, the findings suggest that teamwork and collaboration within and across professional, organizational, technical, and cultural boundaries may be related to the delivery of safe and high‐quality care [71].

The findings from two studies reporting no significant associations between teamwork and call light response time [30], as well as missed care [51], suggest that certain patient outcomes may be influenced by factors beyond team dynamics alone. These findings highlight the complexity of healthcare environments, where outcomes such as call light response time and missed nursing care may be influenced by multiple organizational and contextual factors, including staffing levels, workload, unit characteristics, and resource availability. Therefore, while teamwork appears to be an important factor, its relationship with patient care outcomes may depend on broader workplace conditions.

The findings of this review suggest that positive teamwork attitudes are associated with favorable nurse‐ and patient‐related outcomes in hospital settings. These findings highlight the potential value of promoting teamwork through team training, supportive leadership, and communication‐enhancing strategies. Healthcare organizations and policymakers should consider teamwork as an important component of workforce stability and quality improvement initiatives. Future longitudinal studies and meta‐analyses are needed to further examine the relationships between teamwork and clinical outcomes and to identify approaches for supporting collaborative work environments.

The findings of this study are broadly consistent with the integrative framework and suggest that positive teamwork attitudes are associated with favorable nurse well‐being and patient‐related outcomes [23]. Our results highlight the associations between teamwork and higher nurse job satisfaction, lower burnout, and more favorable patient safety indicators, which are consistent with the proposed relationships within the framework. This evidence highlights the potential importance of collaborative team environments in relation to clinician occupational well‐being and patient safety. The findings suggest that the framework may serve as a useful foundation for future research and the development of interventions aimed at strengthening teamwork and improving healthcare outcomes.

## 5. Recommendations for Future Research

Future research should employ longitudinal, experimental, and other rigorous study designs to further examine the relationships between nurses’ teamwork attitudes and nurse‐ and patient‐related outcomes. Such studies may provide stronger evidence regarding potential causal relationships and underlying mechanisms. Further work is also needed to evaluate and cross‐culturally validate teamwork measurement instruments across diverse clinical settings. In addition, future primary studies and reviews should explore the association between teamwork culture and broader organizational outcomes, such as efficiency and hospital performance indicators. Greater attention should also be given to potential mediating and confounding factors that may influence the relationships between teamwork and outcomes.

### 5.1. Limitations of Study

The present systematic review has several important limitations that should be considered when interpreting the findings. First, the evidence base was predominantly composed of cross‐sectional observational studies, which limits the ability to draw causal inferences and introduces the possibility of reverse causality between nurses’ teamwork and reported outcomes. Second, many included studies relied on self‐reported measures for both teamwork and outcomes, which may introduce common‐method bias and potentially inflate observed associations. In addition, residual confounding cannot be excluded, as most studies did not fully adjust for all relevant organizational and individual factors.

Third, there was considerable heterogeneity in the conceptualization and measurement of teamwork, including constructs such as teamwork attitudes, teamwork climate, collaboration, cohesion, participative teamwork, and perceived teamwork. This variability may limit the comparability of findings across studies and complicate the synthesis of evidence.

Fourth, the methodological heterogeneity of outcome measures and study designs precluded the conduct of a meta‐analysis and limited the ability to quantitatively assess the strength of evidence. Finally, certainty of evidence across outcomes was not formally assessed, which limits the ability to grade the overall confidence in the findings. Therefore, the results should be interpreted cautiously as associative evidence rather than causal relationships. Also, the review protocol was not registered in a public database (e.g., PROSPERO), which may limit transparency.

## 6. Conclusions

The findings of this systematic review suggest that nurses’ perceptions of teamwork are associated with a range of nurse and patient outcomes in hospital settings. These results may provide useful insights for nurses, nurse managers, health policymakers, and nursing educators when considering organizational and educational approaches related to teamwork in clinical practice. However, given the predominance of cross‐sectional studies and self‐reported measures, the findings should be interpreted as evidence of association rather than causation. Further longitudinal and interventional research is needed to clarify the directionality of these relationships, examine potential confounding and mediating factors, and evaluate whether teamwork‐focused initiatives are associated with subsequent changes in nurse and patient outcomes.

## Author Contributions

Edris Kakemam and Farzaneh Miri developed the design of this study. All authors contributed to searching in the database, the selection of the study, data extraction, and data analysis. Farzaneh Miri, Alireza Hajizadeh, Razie Alipour, and Edris Kakemam crafted the first draft of the manuscript. Jalal Saeidpour, Masoud Arefnezhad, and Young Sook Roh fundamentally revised the draft.

## Funding

This study received no external funding.

## Disclosure

All authors checked and approved the revised draft of the manuscript.

## Ethics Statement

There were no specific ethical considerations for this study, as it was based on previously published studies and did not involve direct interaction with human participants or access to identifiable personal data. However, this study was approved by the Ethics Committee of Qazvin University of Medical Sciences (IR.QUMS.REC.1404.092).

## Conflicts of Interest

The authors declare no conflicts of interest.

## Supporting Information

Additional supporting information can be found online in the Supporting Information section.

## Supporting information


**Supporting Information 1** Supporting file 1 consists of search strategies in databases.

**Supporting Information 2** The characteristics of the included studies are presented in Supporting File 2.

## Data Availability

The data that support the findings of this study are available from the corresponding author upon reasonable request.
