# Supplementary material for: The Association Between Nurses’ Perceptions of Teamwork and Nurse and Patient Outcomes in Hospital Settings: A Systematic Review
Source: J Nurs Manag. 2026 Aug 4;2026:3356709. doi: 10.1155/jonm/3356709 (PMC13438861; doi:10.1155/jonm/3356709)
Supplement: Supplementary file 1 [file JONM-2026-3356709-s001.docx]

**Table 2. Characteristics of included studies**

| **First authors (Reference)** | **Year** | **Country** | **Sample size** | **Setting** | **Outcomes** | | **Assessment tool of nurses’ attitude toward teamwork** | **Teamwork score** |
| --- | --- | --- | --- | --- | --- | --- | --- | --- |
|  |  |  |  |  | **Nurse outcomes** | **Patient outcomes** |  |  |
| Estryn-Béhar et all. [1] | 2007 | Ten European countries | 28,561 | Any type of hospitals | Intention to leave | - | Questionnaire developed by NEXT Study Group in Urupa | Not reported |
| Mohr et all [2] | 2008 | USA | 110,490 | Any type of hospitals | Resignation rates in hospital | - | Instrument developed after extensive work and data collection within a prior pilot study of Veterans Health Administration hospitals. | 2.84 out of 5 |
| Kalisch et all [3] | 2010 | USA | 3,675 | General hospitals | Job satisfaction | - | NTS | 1.89 out of 5 |
| Kalisch and Hee Lee [4] | 2010 | USA | 2,216 | General hospitals | - | Missed nursing care | NTS | 3.49 out of 5 |
| Abualrub et al [5] | 2012 | Jordan | 450 | Two teaching hospitals and four private hospitals | Safety climate and Intention to stay | - | Teamwork scale that included 14 items (Sexton et al., 2006) | 3.29 out of 5 |
| Kalisch et al [6] | 2013 | USA | 287 | 3 hospitals | - | Time to respond to call lights of patients | NTS | 3.33 out of 5 |
| Li [7] | 2013 | Taiwan | 407 | A county hospital | Safety attitudes | - | T-TAQ | 4.99 out of 7 |
| Hwang and Ahn [8] | 2015 | South Korea | 674 | Teaching hospital | - | Clinical error reporting | T-TAQ | 3.5 out of 5 |
| Trybou et al [9] | 2015 | Belgium | 217 | Teaching hospital | Job satisfaction, Commitment, and Intention to leave | - | The 9-item scale of Major et al. (1995) | Not reported |
| Kaiser and Westers [10] | 2018 | USA | 1,414 | 11 hospitals | Job satisfaction and plans to leave their position | - | NTS | 3.61 out of 5 |
| Logan and Malone [11] | 2018 | USA | 128 | Medium-sized hospitals | Negative acts/bullying | - | Attitudes About Teamwork Scale (Natvig & Malone, 1992) and Team Characteristics Survey (Malone, 1995) | 3.94 out of 5 |
| Anderson et al [12] | 2019 | UK | 302 | Teaching hospital | - | Perceptions of care quality | NTS | 3.07 out of 5 |
| Celik et al [13] | 2019 | Turkey | 116 | Surgical clinics of a state hospital/ Surgical clinic | - | Caring behaviors | T-TAQ | 4.00 out of 5 |
| Ghezeljeh et al [14] | 2021 | Iran | 213 | Teaching hospital | - | Missed nursing care | NTS | 3.53 out of 5 |
| Kakemam et al [15] | 2021 | Iran | 327 | Teaching hospital | - | Occurrence of AEs and Reporting of AEs | T-TAQ | 3.81 out of 5 |
| Cho et all [16] | 2022 | USA | 810 | Hospital | Workload and Occupational fatigue | - | NTS | Not reported |
| Moussa et al [17] | 2022 | Saudi Arabia | 68 | Public tertiary hospitals | Patient safety attitudes | - | T-TAQ | 3.81 out of 5 |
| Baek et al. [18] | 2023 | South Korea | 992 | 100-1000 beds hospital | - | PCC | NTS | Not reported |
| Bragadóttir et al. [19] | 2023 | Iceland | 567 | Teaching and other hospitals | Job satisfaction | - | NTS | 3.89 out of 5 |
| Nobahar et al. [20] | 2023 | Iran | 200 | 3 teaching hospitals | Moral sensitivity | Missed nursing care | T-TAQ | 3.47 out of 5 |
| Azizi et al. [21] | 2024 | Iran | 375 | Teaching hospital | Moral courage | Safe nursing care | T-TAQ | 4.12 out of 5 |
| Dlamini and Park [22] | 2024 | Eswatini | 123 | Teaching hospitals | - | PCC | T-TAQ | 3.82 out of 5 |
| Soyer Er and Gül [23] | 2024 | Turkey | 117 | Teaching hospitals | Nurses' speaking up for patient safety concerns and Nurses' speaking up for unprofessional behaviors | - | Teamwork Climate dimension of the Safety Attitudes Scale | 3.69 out of 5 |
| Alipour et al. [24] | 2024 | Iran | 190 | Teaching hospitals | Moral distress | - | T-TAQ | 3.73 out of 5 |
| Jia et al. [25] | 2025 | China | 182 | Public hospitals | Moral Sensitivity | Missed nursing Care | T-TAQ | 4.33 out of 5 |
| Tasbihi et al. [26] | 2025 | Iran | 472 | Teaching public hospitals | Patient safety competency | - | T-TAQ | 4.06 out of 6 |
| Habibi Sool et al. [27] | 2022 | Iran | 256 | Five educational hospitals | Patient safety competency | - | T-TAQ | 3.60 of out 5 |
| Han et al. [28] | 2020 | South Korea | 200 | Tertiary or secondary hospitals | Patient safety competency | - | T-TAQ | 3.89 out of 5 |
| Akyüz et al. [29] | 2021 | Turkey | 253 | Public hospital | Conflict with Colleagues and Nurse Job Satisfaction | - | T-TAQ | Not reported |
| Brunault et al. [30] | 2013 | France | 84 | Psychiatric hospital | Quality of work life | - | Questionnaire of participative teamwork items: existence and availability of vocational training, existence of ward staff, and participation in interdisciplinary staff meetings. | 2.6 out of 4 |
| Bae et al. [31] | 2023 | South Korea | 264 | 35 hospitals | Job satisfaction and intent to leave | - | Collaboration between nurses was measured using the nurse–nurse collaboration scale developed by Dougherty and Larson and Team cohesion in the nursing units was measured using the team cohesion scale developed by Carless and De Paola | 2.83 out of 4 |
| Sadeghpour et al. [32] | 2026 | Iran | 401 | 4 public and private hospitals | - | Caring behaviors | T-TAQ | 3.64 out of 5 |
| Salahat and AliSaleh [33] | 2026 | Jordon | 375 | Four governmental sector, private sector, royal medical  services, and university-affiliated hospitals | - | Patient Falls | NTS | 3.5 out of 5 |

NTS: Nursing Teamwork Survey; T-TAQ:TeamSTEPPS Teamwork Attitudes Questionnaire; AEs: Adverse Events; PCC: Patient-Centered Care
