# Supplementary material for: The Association Between Nurses’ Perceptions of Teamwork and Nurse and Patient Outcomes in Hospital Settings: A Systematic Review
Source: J Nurs Manag. 2026 Aug 4;2026:3356709. doi: 10.1155/jonm/3356709 (PMC13438861; doi:10.1155/jonm/3356709)
Supplement: Supplementary file 2 [file JONM-2026-3356709-s002.docx]

**Table 1. Search strategies in databases (English language)**

| Database | SET | STRATEGY | RESULT |
| --- | --- | --- | --- |
| **PubMed** | #1 | "teamwork"[Title/Abstract] OR "interprofessional relations"[Title/Abstract] OR "group work"[Title/Abstract] OR "cooperative behavior"[Title/Abstract] | **21,059** |
|  | #2 | "nurses"[MeSH Terms] OR "nursing staff"[MeSH Terms] | **172,055** |
|  | #3 | #1 AND #2 | 1,546 |
|  | #4 | Filters: from 2000 - 2025 | 1,406 |
| **Scopus** |  | ( TITLE ( teamwork ) OR TITLE ( "interprofessional relations" ) OR TITLE ( "group work" ) OR TITLE ( "cooperative behavior" ) AND TITLE ( "nurses" ) OR TITLE ( "nursing staff" ) ) | 264 |
| **Web of science** |  | (((((TI=("teamwork")) OR TI=("interprofessional relations")) OR TI=("group work")) OR TI=("cooperative behavior")) AND TI=(nurses)) OR TI=("nursing staff") | 2,368 |
| **CINAHL** |  | TI (TEAMWORK) OR TI (INTERPROFESSIONAL RELATIONS) OR TI (GROUP WORK) OR TI (cooperative behavior) AND TI (NURSES) AND TI (NURSING STAFF) | 1,157 |
